# Supplementary material for: UK-based, multisite, prospective cohort study of small bowel obstruction in acute surgical services: National Audit of Small Bowel Obstruction (NASBO) protocol
Source: BMJ Open. 2017 Oct 5;7(10):e016796. doi: 10.1136/bmjopen-2017-016796 (PMC5640021; doi:10.1136/bmjopen-2017-016796)
Supplement: Supplementary file 1 [file bmjopen-2017-016796supp001.pdf]

Appendix 1. Data fields collected for cohort study

Field	Options (definitions)
**Age**	In years
**Height**	In centimetres,
**Weight**	In kilograms
**Sex**	Male, Female
**Comorbidities**	Myocardial infarct<br>Congestive heart failure<br>Peripheral vascular disease<br>Cerebrovascular disease (except hemiplegia)<br>Dementia<br>Chronic pulmonary disease<br>Connective tissue disease<br>Peptic ulcer disease<br>Mild liver disease<br>Diabetes (without complications)<br>Diabetes with end organ damage<br>Hemiplegia (or paraplegia)<br>Moderate or severe renal disease<br>Solid tumour (non-metastatic)<br>Leukaemia<br>Lymphoma, Multiple Myeloma...<br>Moderate or severe liver disease<br>Metastatic solid tumour<br>AIDS
**Source of referral**	Emergency Department<br>General Practice<br>Surgical Clinic admission<br>Referral from inpatient team

**Where was the patient living prior to admission to the hospital?**	Own Home/Sheltered Accommodation<br>Residential Home<br>Nursing Home
**Date admitted to hospital**	Day/Month/Year
**Date first seen by a member of the surgical team**	Day/Month/Year
**Date of last enteral intake**	Day/Month/Year
**Initial management strategy**	Conservative<br>Operative (<br>Palliative
**White Cell Count**	
**C-Reactive Protein**	
**Albumin**	
**Did the patient have an AKI at admission?**	Yes / No
**Was the patient identified as being malnourished, or at risk of malnourishment?**	Yes / No<br>Day/Month/Year
**How was this identified?**	
**Was the patient reviewed by a dietitian or nutrition team during admission?**	Yes / No
**Were oral supplements (e.g. fortisips) started at any point started at any point during admission?**	Yes / No<br>Day/Month/Year
**Was NG or NJ feed started during admission?**	Yes / No<br>Day/Month/Year
**Was TPN started during the admission?**	Yes / No<br>Day/Month/Year
**If TPN was used, when was it stopped?**	Day/Month/Year
**Was intravenous access established for nutrition?**	Yes / No

**What type of line was initially used?**	Peripheral cannula<br>Peripherally inserted central catheter (PICC)<br>Central venous catheter (CVC/Central line)<br>Hickmann line
**What date was this inserted?**	Day/Month/Year
**Did the patient develop line sepsis related to this line?**	Yes / No
**Date line sepsis diagnosed**	Day/Month/Year
**Abdominal X-ray performed**	Yes / No<br>Day/Month/Year
**CT scan performed**	Yes / No<br>Day/Month/Year
**Did the patient receive water-soluble contrast agent (gastrografin) apart from when undergoing a CT scan?**	Yes / No<br>Day/Month/Year
**Aetiology**	Congenital band adhesion<br>Post-operative adhesions<br>Right sided colon cancer<br>Crohn's disease<br>Disseminated intra-abdominal malignancy<br>Incarcerated Hernia - Groin<br>Incarcerated hernia - Midline<br>Incarcerated hernia - Incisional<br>Incarcerated Hernia - Parastomal<br>Small bowel Volvulus<br>Other________________
**Did the patient undergo an operation/procedure for SBO?**	Yes / No<br>Day/Month/Year
**American Society of Anesthesiologist (ASA)**	Classified as:

**score**	1. A normal healthy patient<br>2. A patient with mild systemic disease<br>3. A patient with severe systemic disease<br>4. A patient with severe systemic disease that is a constant threat to life<br>5. A moribund patient who is not expected to survive without the operation
**Method of operation**	Laparoscopic<br>Lap converted to open<br>Open (midline)<br>Open (groin)<br>Open (other)
**What intervention?**	Division (single) band adhesion<br>Adhesiolysis<br>Hernia repair<br>Small bowel resection<br>Large bowel resection<br>Formation jejunostomy<br>Formation ileostomy<br>Anastomosis of bowel<br>Other
**Date resumed enteral nutrition**	Day/Month/Year
**In hospital death**	Yes / No
**Date patient medically fit for discharge:**	Day/Month/Year
**Date of discharge**	Day/Month/Year
**Readmitted within 30-days post discharge**	Yes / No

**Discharge destination**	Own Home/Sheltered Accommodation<br>Rehabilitation Unit<br>Residential Home<br>Nursing Home<br>Hospice<br>Still acute inpatient on 30/4/17<br>Deceased
**In hospital complications**	UTI<br>Pneumonia<br>Cardiac<br>PE/DVT<br>Delirium<br>Superficial surgical site infection<br>Intra abdominal sepsis<br>Abdominal wall dehiscence<br>Anastomotic leak<br>Radiological drain<br>Reoperation<br>Unplanned HDU/ITU admission
