# Supplementary material for: UK-based, multisite, prospective cohort study of small bowel obstruction in acute surgical services: National Audit of Small Bowel Obstruction (NASBO) protocol
Source: BMJ Open. 2017 Oct 5;7(10):e016796. doi: 10.1136/bmjopen-2017-016796 (PMC5640021; doi:10.1136/bmjopen-2017-016796)
Supplement: Author's manuscript [file bmjopen-2017-016796.draft_revisions.pdf]

BMJ Open is committed to open peer review. As part of this commitment we make the peer review history of every article we publish publicly available.

When an article is published we post the peer reviewers' comments and the authors' responses online. We also post the versions of the paper that were used during peer review. These are the versions that the peer review comments apply to.

The versions of the paper that follow are the versions that were submitted during the peer review process. They are not the versions of record or the final published versions. They should not be cited or distributed as the published version of this manuscript.

BMJ Open is an open access journal and the full, final, typeset and author-corrected version of record of the manuscript is available on our site with no access controls, subscription charges or pay-per-view fees (http://bmjopen.bmj.com).

If you have any questions on BMJ Open's open peer review process please email editorial.bmjopen@bmj.com

Study Protocol: The National Audit of Small Bowel Obstruction

Journal:	*BMJ Open*
Manuscript ID	bmjopen-2017-016796
Article Type:	Protocol
Date Submitted by the Author:	10-Mar-2017

Complete List of Authors:	Lee, Matthew; Sheffield Teaching Hospitals NHS FT, Department of General Surgery; South Yorkshire Surgical Research Group<br>Sayers, Adele; Pinderfields General Hospital, General Surgery; South Yorkshire Surgical Research Group<br>Drake, Thomas ; University of Edinburgh, 51 Little France Crescent, Edinburgh, EH16 4SA, United Kingdom, Department of Clinical Surgery, ; South Yorkshire Surgical Research Group<br>Hollyman, Marianne; North Bristol NHS Trust; Severn and Peninsula Audit and Research Collaborative<br>Bradburn, Mike; University of Sheffield School of Health and Related Research, Clinical Trials Research Unit<br>Hind, Daniel; University of Sheffield School of Health and Related Research<br>Wilson, Timothy; Doncaster and Bassetlaw Teaching Hospitals NHS Foundation Trust, General Surgery; Association of Coloproctology of Great Britain and Ireland<br>Fearnhead, Nicola; Addenbrooke's Hospital, Colorectal Surgery; Association of Coloproctology of Great Britain and Ireland<br>Steering Group, NASBO; National Audit of Small Bowel Obstruction

**Primary Subject Heading**:	Surgery
Secondary Subject Heading:	Surgery, Nutrition and metabolism
Keywords:	SURGERY, Nutritional support < GASTROENTEROLOGY, HEALTH SERVICES ADMINISTRATION & MANAGEMENT

SCHOLARONE™
Manuscripts

Study Protocol: The National Audit of Small Bowel Obstruction

MJ Lee[1,2], AE Sayers[2,3], TM Drake[2,4], M Hollyman[5,6], M Bradburn[7], D Hind[7], TR Wilson[8,9], NS Fearnhead[9,10] on behalf of the NASBO Steering Group*

1. Sheffield Teaching Hospitals NHS Foundation Trust, UK, S5 7AU
2. South Yorkshire Surgical Research Group, Sheffield, UK, S5 7AU
3. Mid-Yorkshire NHS Trust, Wakefield, UK, WF1 4DG
4. Department of Clinical Surgery, University of Edinburgh, Edinburgh, UK, EH16 4SA
5. North Bristol NHS Trust, Bristol, UK, BS10 5NB
6. Severn and Peninsula Audit and Research Collaborative, Bristol, UK, BS10 5NB
7. Clinical Trials Research Unit, School of Health and Related Research, Sheffield, UK, S1 4DA
8. Doncaster and Bassetlaw Teaching Hospitals NHS Foundation Trust, Doncaster, UK, DN2 5LT
9. Association of Coloproctology of Great Britain and Ireland, London, UK WC2A 3PE
10. Addenbrooke's Hospital, Cambridge University Hospitals NHS Foundation Trust, UK, CB2 0QQ

*Listed in Appendix 1.

Corresponding author:

For peer review only

Matthew Lee, Clinical Research Fellow, Sheffield Teaching Hospitals NHS Foundation Trust, Sheffield, S5 7AU

Email: m.j.lee@sheffield.ac.uk

Telephone: +44 (0) 114 243 43 43

Fax: +44 (0) 114 243 43 43

Twitter: @NASBO2017 / @wannabehawkeye

For peer review only

Funding:

This project has been funded by the Bowel Disease Research Foundation, Association of Coloproctology of Great Britain and Ireland, Association of Surgeons of Great Britain and Ireland, Association of Upper Gastrointestinal Surgeons, British Association of Parenteral and Enteral Nutrition, British Association for Surgical Oncology, British Society of Gastroenterology, Royal College of Surgeons of England, Royal College of Surgeons of Edinburgh, National Emergency Laparotomy Audit and Royal College of Anaesthetists.

Provenance:

Not commissioned, externally peer-reviewed

Author Contributions:

All authors and collaborators contributed to the development of the protocol for the project. Main drafts of text and revisions undertaken by MJL, AES, TMD, MH, MB, DH, NSF and TRW. All authors including collaborators have reviewed and approved the manuscript.

Conflicts of interest:

The authors have no conflicts of interest to declare

Word count: 1849

Figures: 2

Tables: 1

Keywords:

Small bowel obstruction, surgery, nutrition

For peer review only

ABSTRACT

Introduction

Small bowel obstruction (SBO) is a common indication for emergency laparotomy in the UK, which is associated with a 90-day mortality rate of 13%. There are currently no UK clinical guidelines for the management of this condition. The aim of this multi-centre prospective cohort study is to describe the burden, variation in management and associated outcomes of SBO in the UK adult population.

Methods and analysis

UK hospitals providing emergency general surgery are eligible to participate. This study has three components: i) a clinical preference questionnaire to be completed by consultants providing emergency general surgical care to assesses preferences in diagnostics and therapeutic approaches, including laparoscopy and nutritional interventions; ii) site resource profile questionnaire to indicate ease of access to diagnostic services, operating theatres, nutritional support teams and post-operative support including intensive care; iii) prospective cohort study of all cases of small bowel obstruction admitted during an eight-week period at participating trusts. Data on diagnostics, operative and nutritional interventions, and in-hospital mortality and morbidity will be captured, followed by data validation.

Ethics and dissemination

This will be conducted as a national audit of practice in conjunction with trainee research collaboratives, with support from patient representatives, surgeons, anaesthetists, gastroenterologists and a clinical trials unit. Site-specific reports will

For peer review only

be provided to each participant site as well as an overall report to be disseminated through specialist societies. Results will be published in a formal project report endorsed by stakeholders, and in peer-reviewed scientific reports. Key findings will be debated at a focussed national meeting with a view to quality improvement initiatives.

For peer review only

STRENGTHS AND LIMITATIONS

- This study will be the largest prospective assessment of the management of Small Bowel Obstruction in adults in the UK.
- This study will highlight variation in resources and clinical practice, and assess the impact of variation on patient outcomes.
- The methodology limits data to easily measured key components of the treatment pathway that are routinely captured in patient notes.
- Accuracy of data-collection will be assessed in a short *post hoc* validation exercise.
- Potential inclusion of all hospitals providing emergency general surgery will ensure that findings are broadly representative of UK practice.

For peer review only

BACKGROUND

Mechanical small bowel obstruction (SBO) is a common presentation to emergency general surgery. Eleven and a half thousand patients in England and Wales underwent emergency laparotomy for SBO in In the twelve months from 2014-2015 [1]. This was associated with indication with an associated 90-day mortality rate of 13%[1]. Similar findings have been noted in the United States of America[2].

Small bowel obstruction has several aetiologies, including congenital or post-operative adhesions, abdominal wall hernia and malignancy. Plain film radiography or computer tomography (CT) may be used to confirm the diagnosis and determine underlying cause. Depending on aetiology and comorbidities, patients may be selected for early surgical intervention or conservative management, typically with nasogastric decompression, urinary catheterisation and intravenous fluid therapy[3,4]. Around two thirds of patients managed conservatively for adhesive SBO will settle, but the remainder will require surgery[5] , with a prolonging of the treatment pathway and time to gastrointestinal recovery (Figure 1).

Guidelines already exist in the USA and Europe for the management of SBO[3,4]. The Royal College of Surgeons of England has described a pathway for the management of SBO, although this is presented in guidelines for the commissioning of emergency services, rather than clinical guidelines[6]. This advocates the use of early CT scanning, use of Gastrografin, and timely intervention. Limited specific guidance leads to greater variation in the management of SBO across the UK.

For peer review only

Currently available data do not provide a national overview of the management of SBO: the National Emergency Laparotomy Audit (NELA) captures only the subset of patients who undergo surgery, meaning that we have no high quality information on those managed conservatively and their outcomes[1]. As SBO accounts for half of emergency laparotomies, and likely many more conservatively managed patients, data to inform policy, quality improvement programmes and clinical trials are an audit priority[7],[8].

For peer review only

AIM

The aim of this study is to describe the variation in management and outcomes of SBO in the UK.

Objectives of the study are to describe:

- Variations in consultant practice in the management of SBO
- Variation in resources available to support the management of SBO
- Patient pathways and variation in the management of SBO
- Use of diagnostics in SBO (CT, plain film radiography)
- Interventions used in SBO (operative intervention, therapeutic trial of water soluble contrast agent)
- Use of nutritional assessment tools and resulting nutritional interventions

For peer review only

METHODS

This project has three components: a survey of clinical practice, a site resource questionnaire, and a prospective cohort study (Figure 2). Site recruitment has been undertaken through specialty association conferences and electronic mailing, recruitment presentations at specialty meetings, through trainee research collaboratives, and through professional contacts. All UK hospitals providing emergency general surgery are eligible to participate.

Survey of clinical practice

An anonymous survey of clinical practice has been designed for completion by consultant surgeons providing emergency general surgery care. This captures basic demographic data including specialty and year of graduation. To contextualise clinical data, respondents are asked to indicate the impact of specific clinical factors on the selection of primary operative or conservative management (e.g. multilevel obstruction due to disseminated malignancy, raised or normal inflammatory markers), the minimum investigations required for management, use of Gastrografin, and use of laparoscopy. The survey also investigates preferences around nutritional support in SBO.

Site resource profile

The site resource profile is to be completed once for each participating site. This captures data on staffing levels, ease of access to diagnostics, theatres, and nutritional support teams. This will indicate frequency of handovers of care and

For peer review only - http://bmjopen.bmj.com/site/about/guidelines.xhtml

delays in access to diagnostics: these factors that may delay decision making for these patients. Access to theatres, intensive care, and nutritional support teams will indicate resource for implementing these decisions.

Prospective cohort study

Patients eligible for inclusion in the prospective cohort study must have met the following criteria:

- Have been admitted from the emergency department or primary care to the acute surgery team or referred from an inpatient team to the emergency surgery team
- A clinical diagnosis of SBO made by a specialty trainee year 3 (ST3) or higher in general surgery

These inclusion criteria are purposefully broad with the intention of capturing as many patients with SBO as possible.

Patients will be excluded if:

- They have undergone abdominal surgery within the same hospital admission prior to first symptoms of SBO
- Pregnant women
- Patients under the age of 16 years old
- Patients with large bowel obstruction (even when signs of SBO are present) e.g. obstructing rectal tumour
- Patients with a length of stay <24 hours

For peer review only

Patients will be identified over an eight-week period. Data to be captured include basic demographics, comorbidities in the form of the Charlson Comorbidity Index[9], and usual place of residence (own home, residential home, nursing home) as a proxy for frailty (Appendix 2). Height and weight are captured to calculate Body Mass Index and Nutritional Risk Index as risk adjustment tools[10].

Data will be recorded on initial and final management strategies, baseline physiology, diagnostics and nutritional support strategies.

The primary outcome is in-hospital mortality. Secondary outcomes include in-hospital morbidity, length of stay and 30-day readmission.

Data will be uploaded to an encrypted and password protected secure REDCap server, hosted at the University of Sheffield[11]. No identifiable data is uploaded. Collaborators will keep a local 'key' spreadsheet linking REDCap identifiers to NHS or Hospital Numbers on their NHS network.

Data validation

Only data sets with >95% data completeness will be accepted. Doctors at Core Trainee level or above, who were not involved in initial data collection will act as independent assessors, reviewing data collected at a local centre. Overall independent assessors will validate a minimum of 10% of patient records, with a target of >95% case ascertainment and >98% data accuracy.

For peer review only

The number of identified patients having surgery during the audit period will be compared to those recorded in the NELA database for the same period. This will give an indication of how representative the dataset is.

Pilot

The survey has undergone pilot at two separate sites, with minor revisions after each round.

The prospective audit and site profile questionnaire have undergone a two-week pilot across eight UK centres to confirm acceptability of definitions and usability of REDCap system.

Anticipated recruitment

Based upon NELA data for 2014-2015[1] and pilot work, we anticipate mean identification rates of 3 cases/week per centre. Across 100 centres, anticipated recruitment would be 2,400 cases.

Statistical analysis

Analysis will be performed by a statistician at the Clinical Trials Research Unit, University of Sheffield. Descriptive analysis will be performed to describe raw rates of mortality and morbidity, with sub-group analysis of primary operation, conservative management, and failed conservative management. BMI, Nutritional Risk Index[10], and Charlson Comorbidity Index[9] will be used for risk adjustment. Descriptive reporting of the use of diagnostics, operative approach and nutritional

For peer review only

support in the treatment pathway will be performed, and association with outcomes recorded.

Data will be matched to site resource profiles to assess the relationship between resource availability and management practices.

Ethics and governance

This project has been assessed by the Scientific Officer of the South East Scotland Research Ethics Service, who confirmed that the project did not require ethical approval. All sites must secure local audit approval prior to collecting data, and Information Governance or Caldicott approval prior to uploading data to REDCap. Caldicott approval for Scotland will be secured through a single central application.

Funding

This project has received funding from the Bowel Disease Research Foundation, Association of Coloproctology of Great Britain and Ireland, Association of Surgeons of Great Britain & Ireland, Association of Upper Gastrointestinal Surgeons, British Association of Parenteral and Enteral Nutrition, British Association for Surgical Oncology, British Society of Gastroenterology, Royal College of Surgeons of England, Royal College of Surgeon of Edinburgh, National Emergency Laparotomy Audit and Royal College of Anaesthetists.

Authorship

For peer review only

All collaborators returning complete and validated datasets within the timelines will be eligible for collaborative authorship. This will be reported in line with the CRediT taxonomy[12]. We intend that each site has no more than four collaborators.

For peer review only

DISCUSSION

Small bowel obstruction carries significant morbidity and mortality, however most work on this topic has focussed on specific diagnostic or therapeutic interventions, with little focus on how to address the associated high levels of mortality. The guidance from Eastern Association for the Surgery of Trauma, and World Society for Emergency Surgery offers extensive information on the use of CT scans to identify strangulation or 'high grade' SBO and the selection of patients for surgery (and operative approach), or conservative management[3,4]. This guidance does not substantially address other issues such as nutritional interventions, use of Total Parenteral Nutrition (TPN), or considerations in post-operative care.

This study will deliver the largest prospective assessment of the management of SBO in adults in the UK. Using clinical data on management of SBO, clinician management preferences, and a local resource profile, we will report variation in management of this condition. These data will also permit early exploration of factors associated with variation in practice, and their relationship to outcomes. This study will also provide preliminary data on interventions used in SBO to re-establish feeding. Other studies in the field have focussed only on specific areas of SBO management and to our knowledge, there is very limited data with regards to how nutrition is handled. The central aim of the NASBO project is to address this by delivering high quality data across multiple centres.

For peer review only

This project uses multiple methods to accumulate data including surveys and clinical data collection. Surveys have been carefully designed and piloted to ensure validity and clarity of questions.

The snapshot clinical data-capture has been designed to capture key components of the SBO pathway. Whilst it captures several key nodes of clinical practice, it does not report on the use of nasogastric tubes or use of intravenous fluids. Whilst these are commonly used, accurate data capture to describe them would require a significant amount of resource for what is likely to be highly granular data. If required, these factors could be explored in future studies delivered by the NASBO network. The treatment pathway and pathophysiology of SBO is complex and varied. This complexity, however, must be balanced with the ability to deliver high-quality, usable data. This balance has been emphasised when designing the study and developing data collection tools.

Trainee research collaboratives have previously demonstrated the ability to deliver large multicentre studies[13,14]. This study differs in that it is the first time UK trainee research collaboratives have partnered with a number of specialty organisations and policy makers. The complexity of patient pathways and variation in clinical decision-making make SBO a prime target for intervention. Use and timing of CT, nutritional support and surgical intervention are all potentially costly interventions which are accompanied with risks to the patient. Therefore, it is imperative to generate a high-quality evidence base in a condition which carries a

For peer review only

high mortality and morbidity rate. High quality data on SBO will also allow appropriate assessment of the health economic impact of future interventions.

We envisage this project will allow a network to be formed of clinicians who have an interest in improving outcomes following small bowel obstruction. This network will permit the delivery of quality improvement projects and further, interventional research studies to be performed based upon the results of the inaugural NASBO study.

For peer review only

References

1 National Emergency Laparotomy Audit. The Second Patient Report of the National Emergency Laparotomy Audit (NELA) December 2014 to November 2015. London: 2016.

2 Scott J, Olufajo O, Brat G, *et al.* Use of National Burden to Define Operative Emergency General Surgery. *JAMA Surg* 2016;**151**:e160480.

3 Maung AA, Johnson DC, Piper GL, *et al.* Evaluation and management of small-bowel obstruction. *J Trauma Acute Care Surg* 2012;**73**:S362–9. doi:10.1097/TA.0b013e31827019de

4 Di Saverio S, Coccolini F, Galati M, *et al.* Bologna guidelines for diagnosis and management of adhesive small bowel obstruction (ASBO): 2013 update of the evidence-based guidelines from the world society of emergency surgery ASBO working group. *World J Emerg Surg* 2013;**8**:42. doi:10.1186/1749-7922-8-42

5 Fevang BT, Jensen D, Svanes K, *et al.* Early operation or conservative management of patients with small bowel obstruction? *Eur J Surg* 2002;**168**:475–81. doi:10.1080/110241502321116488

6 ASGBI, Royal College of Surgeons of England. Commissioning guide: Emergency General Surgery. London: 2014.

7 Boney O, Bell M, Bell N, *et al.* Identifying research priorities in anaesthesia and perioperative care: final report of the joint National Institute of Academic Anaesthesia/James Lind Alliance Research Priority Setting Partnership. *BMJ Open* 2015;**5**:e010006. doi:10.1136/bmjopen-2015-010006

8 Tiernan J, Cook A, Geh I, *et al.* Use of a modified Delphi approach to develop

For peer review only

research priorities for the association of coloproctology of Great Britain and Ireland. *Color Dis* 2014;**16**:965–70. doi:10.1111/codi.12790

9 Charlson ME, Pompei P, Ales KL, *et al.* A new method of classifying prognostic comorbidity in longitudinal studies: Development and validation. *J Chronic Dis* 1987;**40**:373–83. doi:10.1016/0021-9681(87)90171-8

10 The Veterans Affairs Total Parenteral Nutrition Cooperative Study Group. Perioperative Total Parenteral Nutrition in Surgical Patients. *N Engl J Med* 1992;**327**:70–5. doi:10.1056/NEJM199207093270202

11 Harris PA, Taylor R, Thielke R, *et al.* Research electronic data capture (REDCap)-A metadata-driven methodology and workflow process for providing translational research informatics support. *J Biomed Inform* 2009;**42**:377–81. doi:10.1016/j.jbi.2008.08.010

12 Brand A, Allen L, Altman M, *et al.* Beyond authorship: Attribution, contribution, collaboration, and credit. *Learn Publ* 2015;**28**:151–5. doi:10.1087/20150211

13 Bhangu A, Richardson C, Torrance A, *et al.* Multicentre observational study of performance variation in provision and outcome of emergency appendicectomy. *Br J Surg* 2013;**100**:1240–52. doi:10.1002/bjs.9201

14 STARSURG Collaborative. Multicentre prospective cohort study of body mass index and postoperative complications following gastrointestinal surgery. *Br J Surg* 2016;:1157–72. doi:10.1002/bjs.10203

For peer review only

Appendix 1: NASBO Steering Group (to be PubMed citable)

John Abercrombie, Austin G Acheson, Derek Alderson, Iain Anderson, Simon Bach, Michael Davies, Zaed Hamady, John Hartley, John Northover, Christopher Lewis, Paul Marriott, Nicholas Maynard, Malcolm McFall, Aravinth Muraganathan, David Murray, Pritam Singh, Gillian Tierney, Azmina Verjee, Ciaran Walsh, Jonathan Wild.

For peer review only

Appendix 2. Data fields collected for cohort study

Field	Options (definitions)
**Age**	In years
**Height**	In centimetres,
**Weight**	In kilograms
**Sex**	Male, Female
**Comorbidities**	Myocardial infarct<br>Congestive heart failure<br>Peripheral vascular disease<br>Cerebrovascular disease (except hemiplegia)<br>Dementia<br>Chronic pulmonary disease<br>Connective tissue disease<br>Peptic ulcer disease<br>Mild liver disease<br>Diabetes (without complications)<br>Diabetes with end organ damage<br>Hemiplegia (or paraplegia)<br>Moderate or severe renal disease<br>Solid tumour (non-metastatic)<br>Leukaemia<br>Lymphoma, Multiple Myeloma...<br>Moderate or severe liver disease<br>Metastatic solid tumour<br>AIDS
**Source of referral**	Emergency Department<br>General Practice<br>Surgical Clinic admission<br>Referral from inpatient team

For peer review only

**Where was the patient living prior to admission to the hospital?**	Own Home/Sheltered Accommodation<br>Residential Home<br>Nursing Home
**Date admitted to hospital**	Day/Month/Year
**Date first seen by a member of the surgical team**	Day/Month/Year
**Date of last enteral intake**	Day/Month/Year
**Initial management strategy**	Conservative<br>Operative (<br>Palliative
**White Cell Count**	
**C-Reactive Protein**	
**Albumin**	
**Did the patient have an AKI at admission?**	Yes / No
**Was the patient identified as being malnourished, or at risk of malnourishment?**	Yes / No<br>Day/Month/Year
**How was this identified?**	
**Was the patient reviewed by a dietitian or nutrition team during admission?**	Yes / No
**Were oral supplements (e.g. fortisips) started at any point started at any point during admission?**	Yes / No<br>Day/Month/Year
**Was NG or NJ feed started during admission?**	Yes / No<br>Day/Month/Year
**Was TPN started during the admission?**	Yes / No<br>Day/Month/Year
**If TPN was used, when was it stopped?**	Day/Month/Year
**Was intravenous access established for nutrition?**	Yes / No

For peer review only

**What type of line was initially used?**	Peripheral cannula<br>Peripherally inserted central catheter (PICC)<br>Central venous catheter (CVC/Central line)<br>Hickmann line
**What date was this inserted?**	Day/Month/Year
**Did the patient develop line sepsis related to this line?**	Yes / No
**Date line sepsis diagnosed**	Day/Month/Year
**Abdominal X-ray performed**	Yes / No<br>Day/Month/Year
**CT scan performed**	Yes / No<br>Day/Month/Year
**Did the patient receive water-soluble contrast agent (gastrografin) apart from when undergoing a CT scan?**	Yes / No<br>Day/Month/Year
**Aetiology**	Congenital band adhesion<br>Post-operative adhesions<br>Right sided colon cancer<br>Crohn's disease<br>Disseminated intra-abdominal malignancy<br>Incarcerated Hernia - Groin<br>Incarcerated hernia - Midline<br>Incarcerated hernia - Incisional<br>Incarcerated Hernia - Parastomal<br>Small bowel Volvulus<br>Other_______________
**Did the patient undergo an operation/procedure for SBO?**	Yes / No<br>Day/Month/Year
**American Society of Anesthesiologist (ASA)**	Classified as:

For peer review only

**score**	1. A normal healthy patient<br>2. A patient with mild systemic disease<br>3. A patient with severe systemic disease<br>4. A patient with severe systemic disease that is a constant threat to life<br>5. A moribund patient who is not expected to survive without the operation
**Method of operation**	Laparoscopic<br>Lap converted to open<br>Open (midline)<br>Open (groin)<br>Open (other)
**What intervention?**	Division (single) band adhesion<br>Adhesiolysis<br>Hernia repair<br>Small bowel resection<br>Large bowel resection<br>Formation jejunostomy<br>Formation ileostomy<br>Anastomosis of bowel<br>Other
**Date resumed enteral nutrition**	Day/Month/Year
**In hospital death**	Yes / No
**Date patient medically fit for discharge:**	Day/Month/Year
**Date of discharge**	Day/Month/Year
**Readmitted within 30-days post discharge**	Yes / No

For peer review only

**Discharge destination**	Own Home/Sheltered Accommodation<br>Rehabilitation Unit<br>Residential Home<br>Nursing Home<br>Hospice<br>Still acute inpatient on 30/4/17<br>Deceased
**In hospital complications**	UTI<br>Pneumonia<br>Cardiac<br>PE/DVT<br>Delirium<br>Superficial surgical site infection<br>Intra abdominal sepsis<br>Abdominal wall dehiscence<br>Anastomotic leak<br>Radiological drain<br>Reoperation<br>Unplanned HDU/ITU admission

For peer review only

Figure headings:

Figure 1: Conceptual schematic of pathways in the management of Small Bowel Obstruction, including typical diagnoses and nutritional outcomes.

Figure 2: Components of NASBO study, and how they are related.

For peer review only

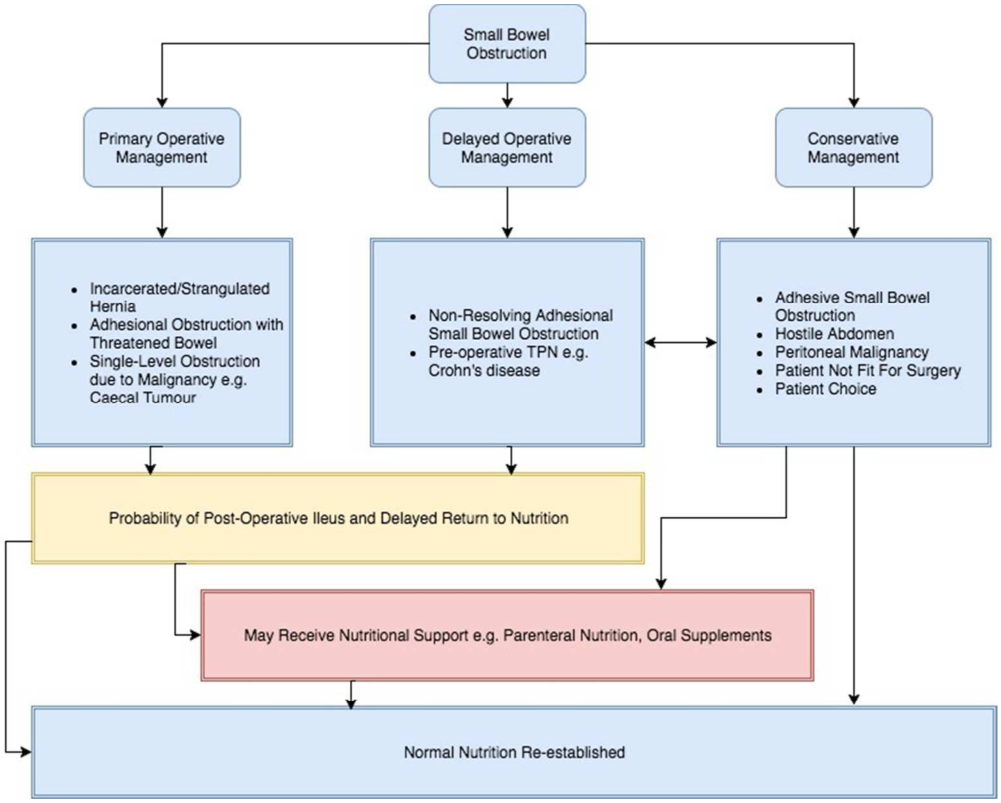

Figure 1: Conceptual schematic of pathways in the management of Small Bowel Obstruction, including typical diagnoses and nutritional outcomes.

270x215mm (72 x 72 DPI)

For peer review only

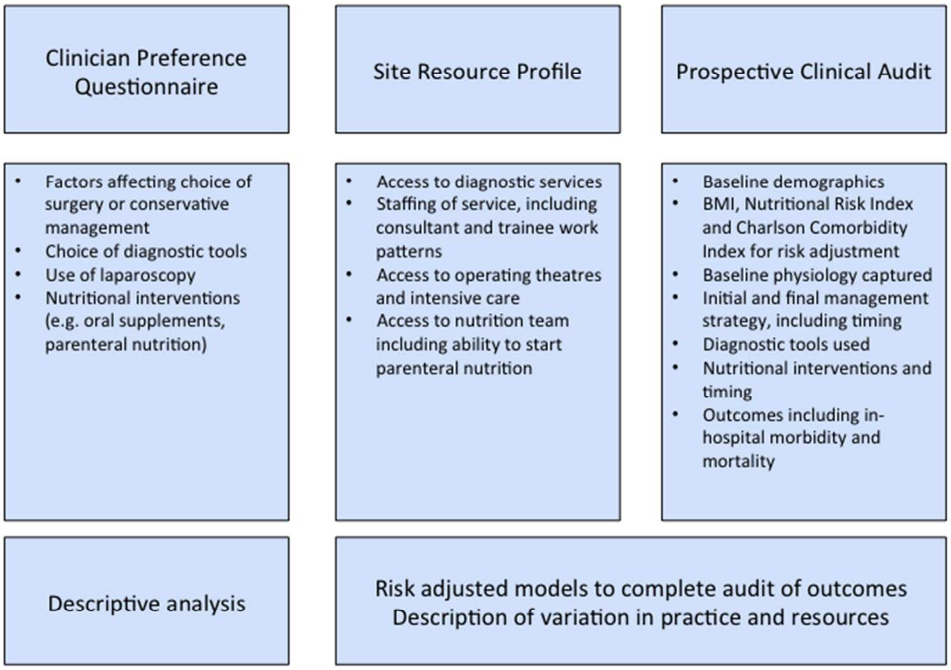

Figure 2: Components of NASBO study, and how they are related.

254x190mm (72 x 72 DPI)

BMJ Open

A UK based, multi-site, prospective cohort study of Small Bowel Obstruction in acute surgical services: National Audit of Small Bowel Obstruction (NASBO) protocol.

Journal:	*BMJ Open*
Manuscript ID	bmjopen-2017-016796.R1
Article Type:	Protocol
Date Submitted by the Author:	08-Aug-2017

Complete List of Authors:	Lee, Matthew; Sheffield Teaching Hospitals NHS FT, Department of General Surgery; South Yorkshire Surgical Research Group<br>Sayers, Adele; Pinderfields General Hospital, General Surgery; South Yorkshire Surgical Research Group<br>Drake, Thomas ; University of Edinburgh, 51 Little France Crescent, Edinburgh, EH16 4SA, United Kingdom, Department of Clinical Surgery, ; South Yorkshire Surgical Research Group<br>Hollyman, Marianne; North Bristol NHS Trust; Severn and Peninsula Audit and Research Collaborative<br>Bradburn, Mike; University of Sheffield School of Health and Related Research, Clinical Trials Research Unit<br>Hind, Daniel; University of Sheffield School of Health and Related Research<br>Wilson, Timothy; Doncaster and Bassetlaw Teaching Hospitals NHS Foundation Trust, General Surgery; Association of Coloproctology of Great Britain and Ireland<br>Fearnhead, Nicola; Addenbrooke's Hospital, Colorectal Surgery; Association of Coloproctology of Great Britain and Ireland<br>Steering Group, NASBO; National Audit of Small Bowel Obstruction

**Primary Subject Heading**:	Surgery
Secondary Subject Heading:	Surgery, Nutrition and metabolism
Keywords:	SURGERY, Nutritional support < GASTROENTEROLOGY, HEALTH SERVICES ADMINISTRATION & MANAGEMENT

SCHOLARONE™ Manuscripts

A UK based, multi-site, prospective cohort study of Small Bowel Obstruction in acute surgical services: National Audit of Small Bowel Obstruction (NASBO) protocol.

MJ Lee[1,2], AE Sayers[2,3], TM Drake[2,4], M Hollyman[5,6], M Bradburn[7], D Hind[7], TR Wilson[8,9], NS Fearnhead[9,10] on behalf of the NASBO Steering Group*

1. Sheffield Teaching Hospitals NHS Foundation Trust, UK, S5 7AU
2. South Yorkshire Surgical Research Group, Sheffield, UK, S5 7AU
3. Mid-Yorkshire NHS Trust, Wakefield, UK, WF1 4DG
4. Department of Clinical Surgery, University of Edinburgh, Edinburgh, UK, EH16 4SA
5. North Bristol NHS Trust, Bristol, UK, BS10 5NB
6. Severn and Peninsula Audit and Research Collaborative, Bristol, UK, BS10 5NB
7. Clinical Trials Research Unit, School of Health and Related Research, Sheffield, UK, S1 4DA
8. Doncaster and Bassetlaw Teaching Hospitals NHS Foundation Trust, Doncaster, UK, DN2 5LT
9. Association of Coloproctology of Great Britain and Ireland, London, UK WC2A 3PE
10. Addenbrooke's Hospital, Cambridge University Hospitals NHS Foundation Trust, UK, CB2 0QQ

*Listed in Supplementary File for Editors

For peer review only

Corresponding author:

Matthew Lee, Clinical Research Fellow, Sheffield Teaching Hospitals NHS Foundation Trust, Sheffield, S5 7AU

Email: m.j.lee@sheffield.ac.uk

Telephone: +44 (0) 114 243 43 43

Fax: +44 (0) 114 243 43 43

Twitter: @NASBO2017 / @wannabehawkeye

For peer review only

Funding:

This project has been funded by the Bowel Disease Research Foundation, Association of Coloproctology of Great Britain and Ireland, Association of Surgeons of Great Britain and Ireland, Association of Upper Gastrointestinal Surgeons, British Association of Parenteral and Enteral Nutrition, British Association for Surgical Oncology, British Society of Gastroenterology, Royal College of Surgeons of England, Royal College of Surgeons of Edinburgh, National Emergency Laparotomy Audit and Royal College of Anaesthetists.

Provenance:

Not commissioned, externally peer-reviewed

Author Contributions:

All authors and collaborators contributed to the development of the protocol for the project. Main drafts of text and revisions undertaken by MJL, AES, TMD, MH, MB,

DH, NSF and TRW. All authors including collaborators have reviewed and approved the manuscript.

Conflicts of interest:

The authors have no conflicts of interest to declare

Word count: 1849

Figures: 2

Tables: 1

Keywords:

Small bowel obstruction, surgery, nutrition

For peer review only

ABSTRACT

Introduction

Small bowel obstruction (SBO) is a common indication for emergency laparotomy in the UK, which is associated with a 90-day mortality rate of 13%. There are currently no UK clinical guidelines for the management of this condition. The aim of this multi-centre prospective cohort study is to describe the burden, variation in management and associated outcomes of SBO in the UK adult population.

Methods and analysis

UK hospitals providing emergency general surgery are eligible to participate. This study has three components: i) a clinical preference questionnaire to be completed by consultants providing emergency general surgical care to assesses preferences in diagnostics and therapeutic approaches, including laparoscopy and nutritional interventions; ii) site resource profile questionnaire to indicate ease of access to diagnostic services, operating theatres, nutritional support teams and post-operative support including intensive care; iii) prospective cohort study of all cases of small bowel obstruction admitted during an eight-week period at participating trusts. Data on diagnostics, operative and nutritional interventions, and in-hospital mortality and morbidity will be captured, followed by data validation.

Ethics and dissemination

For peer review only

This will be conducted as a national audit of practice in conjunction with trainee research collaboratives, with support from patient representatives, surgeons, anaesthetists, gastroenterologists and a clinical trials unit. Site-specific reports will be provided to each participant site as well as an overall report to be disseminated through specialist societies. Results will be published in a formal project report endorsed by stakeholders, and in peer-reviewed scientific reports. Key findings will be debated at a focussed national meeting with a view to quality improvement initiatives.

For peer review only

STRENGTHS AND LIMITATIONS

- This study will be the largest prospective assessment of the management of Small Bowel Obstruction in adults in the UK.
- This study will highlight variation in resources and clinical practice, and assess the impact of variation on patient outcomes.
- The methodology limits data to easily measured key components of the treatment pathway that are routinely captured in patient notes.
- Accuracy of data-collection will be assessed in a short *post hoc* validation exercise.
- Potential inclusion of all hospitals providing emergency general surgery will ensure that findings are broadly representative of UK practice.

For peer review only

BACKGROUND

Mechanical small bowel obstruction (SBO) is a common presentation to emergency general surgery. Eleven and a half thousand patients in England and Wales underwent emergency laparotomy for SBO during the twelve months from April 2014-March 2015 [1]. This was associated with an associated 90-day mortality rate of 13%[1]. Similar findings have been noted in the United States of America[2].

Small bowel obstruction has several aetiologies, including congenital or post-operative adhesions, abdominal wall hernia and malignancy. Plain film radiography or computer tomography (CT) may be used to confirm the diagnosis and determine underlying cause. Depending on aetiology and comorbidities, patients may be selected for early surgical intervention or conservative management, typically with nasogastric decompression, urinary catheterisation and intravenous fluid therapy[3,4]. Around two thirds of patients managed conservatively for adhesive SBO will settle, but the remainder will require surgery[5] , with a prolonging of the treatment pathway and time to gastrointestinal recovery (Figure 1).

Guidelines already exist in the USA and Europe for the management of SBO[3,4]. The Royal College of Surgeons of England has described a pathway for the management of SBO, although this is presented in guidelines for the commissioning of emergency services, rather than clinical guidelines[6]. This advocates the use of early CT scanning, use of Gastrografin, and timely intervention. Limited specific guidance leads to variation in the management of SBO across the UK.

For peer review only

Currently available data do not provide a national overview of the management of SBO: the National Emergency Laparotomy Audit (NELA) captures only the subset of patients who undergo surgery, meaning that we have no high quality information on those managed conservatively and their outcomes[1]. As SBO accounts for half of emergency laparotomies, and likely many more conservatively managed patients, data to inform policy, quality improvement programmes and clinical trials are an audit priority[7],[8].

For peer review only

AIM

The aim of this study is to describe the variation in management and outcomes of SBO in the UK.

Objectives of the study are to describe:

- Variations in consultant practice in the management of SBO
- Variation in resources available to support the management of SBO
- Patient pathways and variation in the management of SBO
- Use of diagnostics in SBO (CT, plain film radiography)
- Interventions used in SBO (operative intervention, therapeutic trial of water soluble contrast agent)
- Use of nutritional assessment tools and resulting nutritional interventions
- Rate of in-hospital mortality in patients treated for SBO
- Rates of 30-day readmission following treatment for SBO
- Rates of unplanned escalation to intensive care

For peer review only

METHODS

This project has three components: a survey of clinical practice, a site resource questionnaire, and a prospective cohort study (Figure 2). Site recruitment has been undertaken through specialty association conferences and electronic mailing, recruitment presentations at specialty meetings, through trainee research collaboratives, and through professional contacts. All UK hospitals providing emergency general surgery are eligible to participate. This project has been registered with the Healthcare Quality Improvement Partnership (HQIP).

Survey of clinical practice

An anonymous survey of clinical practice has been prepared. This is to be completed only by Consultant Surgeons who provide emergency general surgery care – these clinicians are ultimately responsible for the inpatient management of this group and their preferences should influence care rather than other grades of doctor or other specialties. This captures basic demographic data including specialty and year of graduation. To contextualise clinical data, respondents are asked to indicate the impact of specific clinical factors on the selection of primary operative or conservative management (e.g. multilevel obstruction due to disseminated malignancy, raised or normal inflammatory markers), the minimum investigations required for management, use of Gastrografin, and use of laparoscopy. The survey also investigates preferences around nutritional support in SBO. Based on previous experience of surveying surgeons in areas with limited guidance, concerns have been expressed about providing responses out of line with the majority of the profession.

For peer review only

In order to maximise returns, we decided to make this anonymous. This means that we cannot link back to institutions.

Site resource profile

The site resource profile is to be completed once for each participating site. This captures data on staffing levels, ease of access to diagnostics, theatres, and nutritional support teams. This will indicate frequency of handovers of care and delays in access to diagnostics: these factors that may delay decision making for these patients. Access to theatres, intensive care, and nutritional support teams will indicate resource for implementing these decisions. The questionnaire also assesses availability of resources on weekdays, weekends and overnight.

Prospective cohort study

Patients eligible for inclusion in the prospective cohort study must have met the following criteria:

- Have been admitted from the emergency department or primary care to the acute surgery team or referred from an inpatient team to the emergency surgery team
- A clinical diagnosis of SBO made by a specialty trainee year 3 (ST3) or higher in general surgery

These inclusion criteria are purposefully broad with the intention of capturing as many patients with SBO as possible.

Patients will be excluded if:

For peer review only

- They have undergone abdominal surgery within the same hospital admission prior to first symptoms of SBO
- Pregnant women
- Patients under the age of 16 years old
- Patients with large bowel obstruction (even when signs of SBO are present) e.g. obstructing rectal tumour
- Patients with a length of stay <24 hours (discharged home)

Where the initial diagnosis changes, patients will be excluded retrospectively. Patients will be identified over an eight-week period. This period has been selected based on pilot data and NELA reports – to ensure a representative sample of cases and facilitate meaningful analysis, we set a target of 1,500 cases. Extrapolation of numbers from a multi-site pilot suggested that >2,000 cases would be identified during a two week period, with an exclusion rate of around 20%. Consideration was also given to rotation of junior medical staff, who undertake the majority of data collection, and the period avoids most rotation dates. Data to be captured include basic demographics, comorbidities in the form of the Charlson Comorbidity Index[9], and usual place of residence (own home, residential home, nursing home) as a proxy for frailty (Appendix 1). Height and weight are captured to calculate Body Mass Index and Nutritional Risk Index as risk adjustment tools[10].

For peer review only

Data will be recorded on initial and final management strategies, baseline physiology, diagnostics and nutritional support strategies.

The primary outcome is in-hospital mortality. Secondary outcomes include in-hospital morbidity, length of stay and 30-day readmission.

Data will be uploaded to an encrypted and password protected secure REDCap server, hosted at the University of Sheffield[11]. No identifiable data is uploaded. Collaborators will keep a local 'key' spreadsheet linking REDCap identifiers to NHS or Hospital Numbers on their NHS network.

Data validation

Only data sets with >95% data completeness will be accepted. Doctors at Core Trainee level or above, who were not involved in initial data collection will act as independent assessors, reviewing data collected at a local centre. Overall independent assessors will validate a minimum of 10% of patient records, with a target of >95% case ascertainment and >90% data accuracy.

The number of identified patients having surgery during the audit period will be compared to those recorded in the NELA database for the same period. This will give an indication of how representative the dataset is.

Pilot

For peer review only

The survey has undergone pilot at two separate sites, with minor revisions after each round.

The prospective audit and site profile questionnaire have undergone a two-week pilot across eight UK centres to confirm acceptability of definitions and usability of REDCap system.

Anticipated recruitment

Based upon NELA data for 2014-2015[1] and pilot work, we anticipate mean identification rates of 3 cases/week per centre. Across 100 centres, anticipated recruitment would be 2,400 cases.

Statistical analysis

Analysis will be performed by a statistician at the Clinical Trials Research Unit, University of Sheffield. Descriptive analysis will be performed to describe crude rates of mortality and morbidity, with sub-group analysis of primary operation, conservative management, and failed conservative management. BMI, Nutritional Risk Index[10], and Charlson Comorbidity Index[9] will be used for risk adjustment. Descriptive reporting of the use of diagnostics, operative approach and nutritional support in the treatment pathway will be performed, and association with outcomes recorded.

Variation in patient characteristics were taken into account during study design and will be taken into account during statistical analysis. Due to the expected

For peer review only

heterogeneity across all patients, only clinically valid comparisons will be made according to the care pathways outlined in figure 1 (i.e. initial operative management, successful conservative management or failed conservative management). During statistical analysis, multilevel modelling will allow differences across centres to be taken into account. Multilevel logistic regression models will be constructed using clinically plausible variables to identify predictors of mortality and morbidity following small-bowel obstruction. Effects of predictor variables will be presented as odds ratios (OR), alongside the corresponding 95% confidence interval. Sensitivity analyses stratified by number of cases per centre (in the case where hospitals have fewer than 5 cases) will be performed to assess identify any changes to the direction and effect size which may be influenced by the inclusion of centres with few cases.

Data will be matched to site resource profiles to assess the relationship between resource availability and management practices.

Ethics and governance

This project has been assessed by the Scientific Officer of the South East Scotland Research Ethics Service, who confirmed that the project did not require ethical approval. All sites must secure local audit approval prior to collecting data, and Information Governance or Caldicott approval prior to uploading data to REDCap. Caldicott approval for Scotland will be secured through a single central application.

Funding

For peer review only - http://bmjopen.bmj.com/site/about/guidelines.xhtml

This project has received funding from the Bowel Disease Research Foundation, Association of Coloproctology of Great Britain and Ireland, Association of Surgeons of Great Britain & Ireland, Association of Upper Gastrointestinal Surgeons, British Association of Parenteral and Enteral Nutrition, British Association for Surgical Oncology, British Society of Gastroenterology, Royal College of Surgeons of England, Royal College of Surgeon of Edinburgh, National Emergency Laparotomy Audit and Royal College of Anaesthetists.

Authorship

All collaborators returning complete and validated datasets within the timelines will be eligible for collaborative authorship. This will be reported in line with the CRediT taxonomy[12]. We intend that each site has no more than four collaborators.

For peer review only

DISCUSSION

Small bowel obstruction carries significant morbidity and mortality, however most work on this topic has focussed on specific diagnostic or therapeutic interventions, with little focus on how to address the associated high levels of mortality. The guidance from Eastern Association for the Surgery of Trauma, and World Society for Emergency Surgery offers extensive information on the use of CT scans to identify strangulation or 'high grade' SBO and the selection of patients for surgery (and operative approach), or conservative management[3,4]. This guidance does not substantially address other issues such as nutritional interventions, use of Total Parenteral Nutrition (TPN), or considerations in post-operative care.

This study will deliver the largest prospective assessment of the management of SBO in adults in the UK. Using clinical data on management of SBO, clinician management preferences, and a local resource profile, we will report variation in management of this condition. These data will also permit early exploration of factors associated with variation in practice, and their relationship to outcomes. This study will also provide preliminary data on interventions used in SBO to re-establish feeding. Other studies in the field have focussed only on specific areas of SBO management and to our knowledge, there is very limited data with regards to how nutrition is handled. The central aim of the NASBO project is to address this by delivering high quality data across multiple centres.

For peer review only

This project uses multiple methods to accumulate data including surveys and clinical data collection. Surveys have been carefully designed and piloted to ensure validity and clarity of questions.

The snapshot clinical data-capture has been designed to capture key components of the SBO pathway. Whilst it captures several key nodes of clinical practice, it does not report on the use of nasogastric tubes or use of intravenous fluids. Whilst these are commonly used, accurate data capture to describe them would require a significant amount of resource for what is likely to be highly granular data. If required, these factors could be explored in future studies delivered by the NASBO network. The treatment pathway and pathophysiology of SBO is complex and varied. This complexity, however, must be balanced with the ability to deliver high-quality, usable data. This balance has been emphasised when designing the study and developing data collection tools.

Trainee research collaboratives have previously demonstrated the ability to deliver large multicentre studies[13,14]. This study differs in that it is the first time UK trainee research collaboratives have partnered with a number of specialty organisations and policy makers. The complexity of patient pathways and variation in clinical decision-making make SBO a prime target for intervention. Use and timing of CT, nutritional support and surgical intervention are all potentially costly interventions which are accompanied with risks to the patient. Therefore, it is imperative to generate a high-quality evidence base in a condition which carries a high mortality and morbidity rate. High quality data on SBO will also allow

For peer review only

appropriate assessment of the health economic impact of future interventions. Findings of this study will be used to inform development of clinical guidelines, quality indicators, and support development of clinical trials in the field.

We envisage this project will allow a network to be formed of clinicians who have an interest in improving outcomes following small bowel obstruction. This network will permit the delivery of quality improvement projects and further, interventional research studies to be performed based upon the results of the inaugural NASBO study.

For peer review only

References

1 National Emergency Laparotomy Audit. The Second Patient Report of the National Emergency Laparotomy Audit (NELA) December 2014 to November 2015. London: 2016.

2 Scott J, Olufajo O, Brat G, *et al.* Use of National Burden to Define Operative Emergency General Surgery. *JAMA Surg* 2016;**151**:e160480.

3 Maung AA, Johnson DC, Piper GL, *et al.* Evaluation and management of small-bowel obstruction. *J Trauma Acute Care Surg* 2012;**73**:S362–9. doi:10.1097/TA.0b013e31827019de

4 Di Saverio S, Coccolini F, Galati M, *et al.* Bologna guidelines for diagnosis and management of adhesive small bowel obstruction (ASBO): 2013 update of the evidence-based guidelines from the world society of emergency surgery ASBO working group. *World J Emerg Surg* 2013;**8**:42. doi:10.1186/1749-7922-8-42

5 Fevang BT, Jensen D, Svanes K, *et al.* Early operation or conservative management of patients with small bowel obstruction? *Eur J Surg* 2002;**168**:475–81. doi:10.1080/110241502321116488

6 ASGBI, Royal College of Surgeons of England. Commissioning guide: Emergency General Surgery. London: 2014.

7 Boney O, Bell M, Bell N, *et al.* Identifying research priorities in anaesthesia and perioperative care: final report of the joint National Institute of Academic Anaesthesia/James Lind Alliance Research Priority Setting Partnership. *BMJ Open* 2015;**5**:e010006. doi:10.1136/bmjopen-2015-010006

8 Tiernan J, Cook A, Geh I, *et al.* Use of a modified Delphi approach to develop

For peer review only

research priorities for the association of coloproctology of Great Britain and Ireland. *Color Dis* 2014;**16**:965–70. doi:10.1111/codi.12790

9 Charlson ME, Pompei P, Ales KL, *et al.* A new method of classifying prognostic comorbidity in longitudinal studies: Development and validation. *J Chronic Dis* 1987;**40**:373–83. doi:10.1016/0021-9681(87)90171-8

10 The Veterans Affairs Total Parenteral Nutrition Cooperative Study Group. Perioperative Total Parenteral Nutrition in Surgical Patients. *N Engl J Med* 1992;**327**:70–5. doi:10.1056/NEJM199207093270202

11 Harris PA, Taylor R, Thielke R, *et al.* Research electronic data capture (REDCap)-A metadata-driven methodology and workflow process for providing translational research informatics support. *J Biomed Inform* 2009;**42**:377–81. doi:10.1016/j.jbi.2008.08.010

12 Brand A, Allen L, Altman M, *et al.* Beyond authorship: Attribution, contribution, collaboration, and credit. *Learn Publ* 2015;**28**:151–5. doi:10.1087/20150211

13 Bhangu A, Richardson C, Torrance A, *et al.* Multicentre observational study of performance variation in provision and outcome of emergency appendicectomy. *Br J Surg* 2013;**100**:1240–52. doi:10.1002/bjs.9201

14 STARSURG Collaborative. Multicentre prospective cohort study of body mass index and postoperative complications following gastrointestinal surgery. *Br J Surg* 2016;:1157–72. doi:10.1002/bjs.10203

For peer review only

For peer review only

Figure headings:

Figure 1: Conceptual schematic of pathways in the management of Small Bowel Obstruction, including typical diagnoses and nutritional outcomes.

Figure 2: Components of NASBO study, and how they are related.

For peer review only - http://bmjopen.bmj.com/site/about/guidelines.xhtml

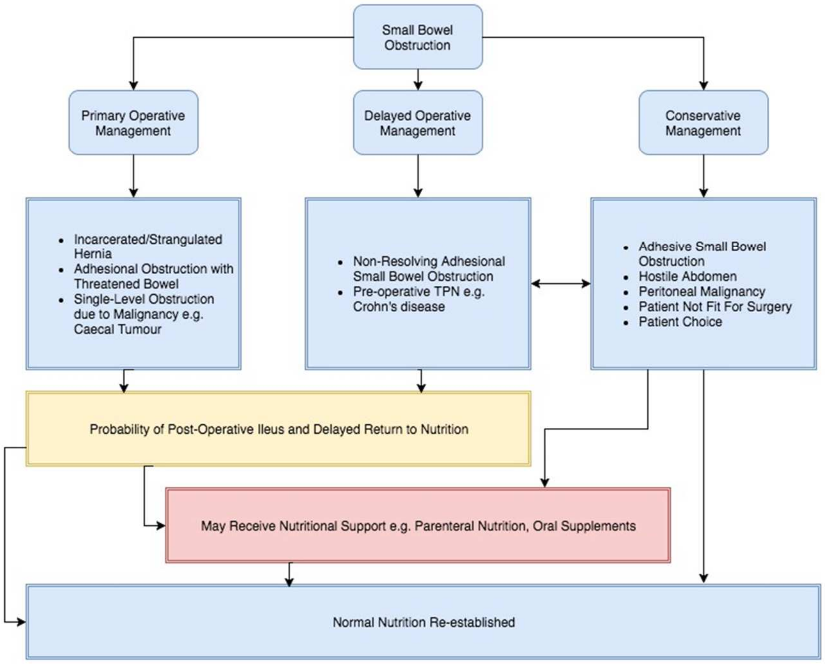

Figure 1: Conceptual schematic of pathways in the management of Small Bowel Obstruction, including typical diagnoses and nutritional outcomes.

78x60mm (300 x 300 DPI)

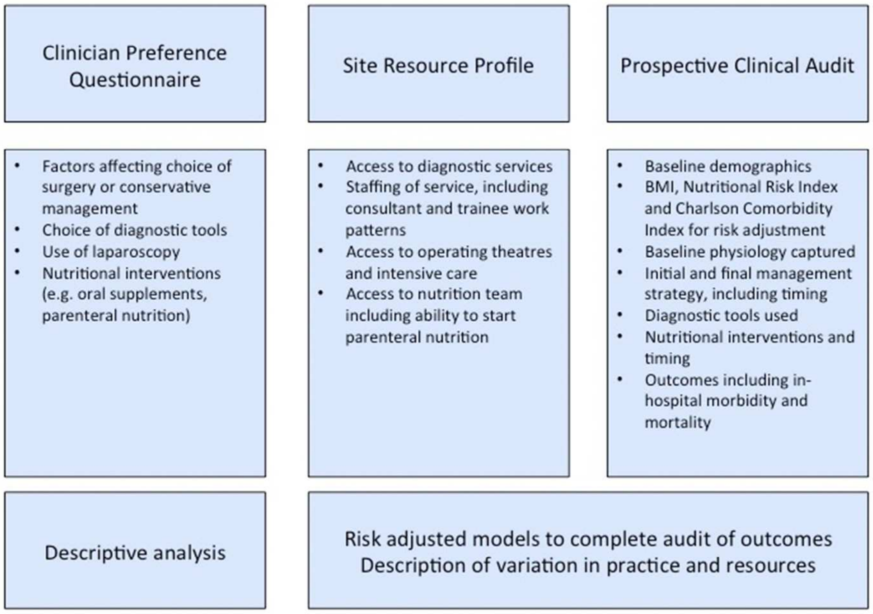

Figure 2: Components of NASBO study, and how they are related.

66x47mm (300 x 300 DPI)

Appendix 1. Data fields collected for cohort study

**Field**	Options (definitions)
**Age**	In years
**Height**	In centimetres,
**Weight**	In kilograms
**Sex**	Male, Female
**Comorbidities**	Myocardial infarct<br>Congestive heart failure<br>Peripheral vascular disease<br>Cerebrovascular disease (except hemiplegia)<br>Dementia<br>Chronic pulmonary disease<br>Connective tissue disease<br>Peptic ulcer disease<br>Mild liver disease<br>Diabetes (without complications)<br>Diabetes with end organ damage<br>Hemiplegia (or paraplegia)<br>Moderate or severe renal disease<br>Solid tumour (non-metastatic)<br>Leukaemia<br>Lymphoma, Multiple Myeloma...<br>Moderate or severe liver disease<br>Metastatic solid tumour<br>AIDS
**Source of referral**	Emergency Department<br>General Practice<br>Surgical Clinic admission<br>Referral from inpatient team

For peer review only

**Where was the patient living prior to admission to the hospital?**	Own Home/Sheltered Accommodation<br>Residential Home<br>Nursing Home
**Date admitted to hospital**	Day/Month/Year
**Date first seen by a member of the surgical team**	Day/Month/Year
**Date of last enteral intake**	Day/Month/Year
**Initial management strategy**	Conservative<br>Operative (<br>Palliative
**White Cell Count**	
**C-Reactive Protein**	
**Albumin**	
**Did the patient have an AKI at admission?**	Yes / No
**Was the patient identified as being malnourished, or at risk of malnourishment?**	Yes / No<br>Day/Month/Year
**How was this identified?**	
**Was the patient reviewed by a dietitian or nutrition team during admission?**	Yes / No
**Were oral supplements (e.g. fortisips) started at any point started at any point during admission?**	Yes / No<br>Day/Month/Year
**Was NG or NJ feed started during admission?**	Yes / No<br>Day/Month/Year
**Was TPN started during the admission?**	Yes / No<br>Day/Month/Year
**If TPN was used, when was it stopped?**	Day/Month/Year
**Was intravenous access established for nutrition?**	Yes / No

For peer review only

**What type of line was initially used?**	Peripheral cannula<br>Peripherally inserted central catheter (PICC)<br>Central venous catheter (CVC/Central line)<br>Hickmann line
**What date was this inserted?**	Day/Month/Year
**Did the patient develop line sepsis related to this line?**	Yes / No
**Date line sepsis diagnosed**	Day/Month/Year
**Abdominal X-ray performed**	Yes / No<br>Day/Month/Year
**CT scan performed**	Yes / No<br>Day/Month/Year
**Did the patient receive water-soluble contrast agent (gastrografin) apart from when undergoing a CT scan?**	Yes / No<br>Day/Month/Year
**Aetiology**	Congenital band adhesion<br>Post-operative adhesions<br>Right sided colon cancer<br>Crohn's disease<br>Disseminated intra-abdominal malignancy<br>Incarcerated Hernia - Groin<br>Incarcerated hernia - Midline<br>Incarcerated hernia - Incisional<br>Incarcerated Hernia - Parastomal<br>Small bowel Volvulus<br>Other_______________
**Did the patient undergo an operation/procedure for SBO?**	Yes / No<br>Day/Month/Year
**American Society of Anesthesiologist (ASA)**	Classified as:

For peer review only

**score**	1. A normal healthy patient<br>2. A patient with mild systemic disease<br>3. A patient with severe systemic disease<br>4. A patient with severe systemic disease that is a constant threat to life<br>5. A moribund patient who is not expected to survive without the operation
**Method of operation**	Laparoscopic<br>Lap converted to open<br>Open (midline)<br>Open (groin)<br>Open (other)
**What intervention?**	Division (single) band adhesion<br>Adhesiolysis<br>Hernia repair<br>Small bowel resection<br>Large bowel resection<br>Formation jejunostomy<br>Formation ileostomy<br>Anastomosis of bowel<br>Other
**Date resumed enteral nutrition**	Day/Month/Year
**In hospital death**	Yes / No
**Date patient medically fit for discharge:**	Day/Month/Year
**Date of discharge**	Day/Month/Year
**Readmitted within 30-days post discharge**	Yes / No

For peer review only

**Discharge destination**	Own Home/Sheltered Accommodation<br>Rehabilitation Unit<br>Residential Home<br>Nursing Home<br>Hospice<br>Still acute inpatient on 30/4/17<br>Deceased
**In hospital complications**	UTI<br>Pneumonia<br>Cardiac<br>PE/DVT<br>Delirium<br>Superficial surgical site infection<br>Intra abdominal sepsis<br>Abdominal wall dehiscence<br>Anastomotic leak<br>Radiological drain<br>Reoperation<br>Unplanned HDU/ITU admission

For peer review only
